# Biochemical and blood gas parameters in llamas (*Lama glama*) raised at moderate altitude: preliminary data from a subtropical environment

**DOI:** 10.1007/s11250-026-05118-2

**Published:** 2026-06-04

**Authors:** Ana Letícia Rodrigues Marques, Marina Marangoni, Vitor Eduardo Mamgue, Paulo Henrique Braz

**Affiliations:** 1 Post-Graduation Program in Health, Welfare and Sustainable Animal Production in the Southern Frontier, Federal University of the Fronteira Sul, Edmundo Gaievski Avenue, 1000, Highway BR 182 - Km 466, PO Box 253, Rural Area, Realeza, PR 85770-000 Brazil; 2 Federal University of the Fronteira Sul, Realeza, Paraná Brazil

**Keywords:** Camelids, Hematology, Parameters

## Abstract

This study presents preliminary baseline biochemical and venous blood gas data obtained from llamas (*Lama glama*) raised under semi-intensive management at medium altitude (595 m asl) in subtropical Brazil. Blood samples from 9 clinically healthy llamas were analyzed. Most biochemical values fell within ranges previously described for the species, although substantial inter-individual variability was observed for several parameters (CK: CV = 0.81; lactate: CV = 0.63; alkaline phosphatase: CV = 0.51; lipids: CV = 0.54), which limits the representativeness of the reported means. Variations in ALT, AST, and CK (200 ± 163 U/L) enzyme levels possibly related to partial confinement and handling was observed. High glucose concentrations (146 ± 14 mg/dL) may reflect species-specific metabolic characteristics, though this interpretation requires validation in larger samples. Urea (46 ± 10 mg/dL) and creatinine (2.16 ± 0.42 mg/dL) reflected dietary protein intake and the species’ efficient nitrogen metabolism. Mineral analysis revealed slightly elevated phosphorus and magnesium, likely due to high-quality forage. Blood gas analysis indicated a lower venous pH (7.269 ± 0.075) and elevated pCO₂ (52.5 ± 1.5 mmHg) and bicarbonate compared to previous reports, potentially consistent with responses to the local environmental conditions. Lactate concentrations (78.9 ± 28 mg/dL) were also elevated; however, this finding should be interpreted cautiously due to potential influences of handling stress, analytical methodology, and sample size limitations. These findings offer valuable baseline data for llamas raised outside their native Andean environment and emphasize the importance of considering environmental and management factors in clinical evaluations. Further studies should include larger populations, arterial blood gas analysis, and longitudinal sampling to better understand physiological adaptations in non-native settings.

## Introduction

Llamas (*Lama glama*) are South American camelids that have been domesticated for thousands of years, primarily in the Andean regions of Peru, Bolivia, Chile, and Argentina (Marín et al. 2007; Miranda-de la Lama and Villarroel 2023). These animals have developed physiological adaptations to high-altitude environments, where hypoxia, low temperatures, and limited nutritional resources impose significant selective pressures (Miranda-de la Lama and Villarroel 2023; Yacobaccio and Vilá 2016).

In recent decades, llamas have been introduced to various regions worldwide, including temperate and low-altitude environments. Camelid farming is still emerging, with increasing interest in their use for exhibitions, ornamental purposes, and ecological grazing management (Marín et al. 2007; Yacobaccio and Vilá 2016). These interest lead to a rise in the diagnosis of nutritional, parasitic, and infectious diseases in these animals, underscores the need for specialized veterinary care and early diagnosis of diseases (Mamgue et al. 2025; Van Saun 2006; Wagener et al. 2024). The alterations detection through biochemical and blood gas exams is crucial for effective disease management and prevention, ensuring the health and productivity of llama populations (Neubert et al. 2021).

Previous studies have established reference values for biochemical parameters in camelids from high-altitude regions (Fowler and Bravo 2010; Fowler and Zinkl 1989). However, there is a lack of specific data for animals raised in different climatic and geographic conditions, such as the temperate climate and medium altitudes.

The establishment of regional reference intervals is essential for improving the clinical evaluation and health management of camelids in non-native environments. Physiological parameters can be influenced by a wide range of factors, including altitude, climate, diet, and management practices (Fowler and Bravo 2010). Studies in other domestic species have demonstrated significant variations in biochemical markers due to environmental and husbandry differences (Abdel Aal et al. 2025; Agradi et al. 2022). Given the increasing number of camelids being raised, it is essential to determine whether reference values established are applicable to animals raised under different conditions.

Thus, this study aims to establish reference intervals parameters in llamas raised in the Southwest region of Paraná, Brazil, determine serum biochemical values and assess venous blood gas parameters in llamas raised under these conditions. These findings contribute to veterinary medicine, animal production, and conservation efforts by providing fundamental baseline data for llamas raised in temperate climates. This information are valuable for veterinarians, researchers, and farmers in diagnosing diseases, monitoring health status, and optimizing management practices, supporting sustainable breeding practices.

## Materials and methods

This study was conducted following ethical guidelines for animal research, ensuring minimal distress to the animals during handling and sample collection. All procedures were performed by trained veterinarians and in accordance with national and international animal welfare regulations. The study was approved by the institutional ethics committee (number 3749290824).

The animals were provide of a private farm in the municipality of Francisco Beltrão, state of Paraná, Brazil (26°05′13.7″ S, 53°07′19.2″ W), at an altitude of 595 m above sea level (m asl). The region has a humid subtropical climate (Köppen classification Cfa), characterized by well-defined seasons, mean annual temperature of approximately 18 °C, and mean annual rainfall of 1,800-2,200 mm. Sampling was conducted during the winter period (August), when ambient temperatures ranged from 8 °C to 20 °C.

The study population comprised 9 clinically healthy llamas, both sexes, aged 1 to 5 years, raised under a semi-intensive management system. Animals were grazing on *Panicum maximum* cv. Aruana and *Cynodon dactylon* (Florakirk) pastures and received supplemental wheat and oat grains ad libitum in feed troughs. No feed restriction (fasting) was imposed prior to blood collection, consistent with standard farm management and with the aim of reflecting routine clinical conditions.

Before sample collection, all animals underwent a complete clinical examination to ensure they were in good health. Anamnesis was conducted through interviews with the farm responsible for their care. This step involved obtaining information regarding the animals’ origin, feeding regimen, reproductive history, past illnesses, and previous medical treatments.

### Blood sample collection and processing

Blood samples were collected in the morning (between 08:00 and 10:00 h) to minimize diurnal variation. Animals were individually restrained by two trained handlers using manual neck restraint; no chemical sedation was employed. The duration of physical restraint from initial contact to completion of venipuncture did not exceed 5 min per animal. Blood was collected from the jugular vein following local trichotomy and antisepsis with 70% ethanol, using a sterile 12 × 8 mm needle attached to a 10 mL syringe. Immediately after sampling, the blood was transferred into serum separator tubes and maintained at 2**–**8 °C until centrifugation and analysis, performed within **3** hours of collection.

Serum biochemical analyses were carried out using an automated chemistry analyzer Wiener lab. CM 250^®^, internal quality control was performed using commercial control sera at two concentration levels (low and high) prior to the analytical run, with acceptable limits defined as a coefficient of variation below 5% for each parameter. The parameters evaluated included uric acid (Uric-A), albumin (Alb), alanine aminotransferase (ALT), aspartate aminotransferase (AST), calcium (Ca), creatine kinase (CK), total cholesterol (Chol), creatinine (Crea), alkaline phosphatase (ALP), iron (Fe), gamma-glutamyl transferase (GGT), lipides (LIP), plasmatic protein (PP), triglycerides (Trig), urea, amylase (Amyl), phosphorus (P), high-density lipoprotein (HDL), lactate (Lac) and magnesium (Mag).

Venous blood gas analysis was performed immediately after collection using the Epoc^®^ Blood Gas Analyzer (Heska Corporation, Loveland, CO, USA), a portable, point-of-care device. The analysis was conducted according to the manufacturer’s recommendations to ensure rapid and accurate assessment. The following parameters were measured: pH, partial pressure of carbon dioxide (pCO₂), bicarbonate (cHCO₃⁻), base excess in the extracellular fluid (BE[ecf]), base excess in the blood (BE[b]), oxygen saturation (cSO₂), sodium (Na⁺), chloride (Cl⁻), total carbon dioxide (TCO₂), glucose (Glu), lactate (Lac), blood urea nitrogen (BUN), and creatinine (Crea).

### Descriptive statistical analysis

Data intervals for biochemical and blood gas parameters were determined following the guidelines outlined by the American Society for Veterinary Clinical Pathology (ASVCP) for establishing reference ranges in veterinary species. The statistical methodology included evaluating the distribution of data, identifying potential outliers, and applying nonparametric statistical methods for determining reference intervals when necessary (Friedrichs et al. 2012).

**Fig. 1:**
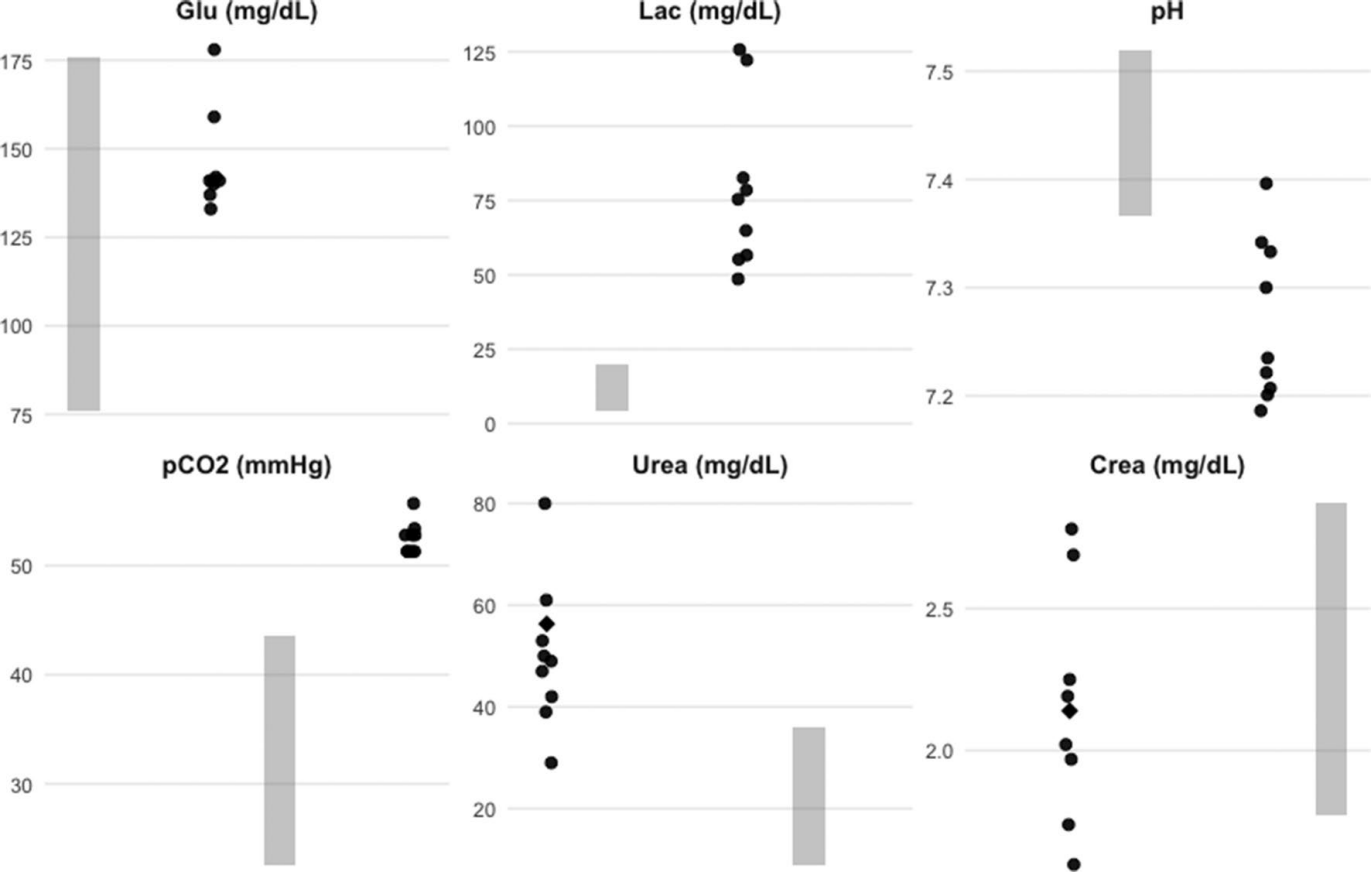
Distribution of key biochemical and venous blood gas parameters in clinically healthy llamas (*Lama glama*) raised under semi-intensive management at medium altitude (595 m) in southern Brazil. Note: Individual data points represent measurements from each animal (*n* = 9). Shaded areas indicate consolidated reference intervals derived from published studies (Fowler and Bravo 2010; Zaki et al. 2023; Viesselmann et al. 2018), and diamonds represent mean values reported in the literature

To facilitate the interpretation of key biochemical and venous blood gas parameters, a graphical comparison with previously published data was performed (Fig. 1). Reference intervals and mean values were extracted from studies reporting biochemical and blood gas parameters in llamas raised under different environmental conditions (Fowler and Bravo 2010; Zaki et al. 2023; Viesselmann et al. 2018).

## Results

A total of 9 adult llamas (aged 1 to 5 years) raised under semi-intensive management in southwestern Paraná, Brazil, were evaluated. Results are presented as means ± standard deviation (Tables 1 and 2) and should be interpreted as preliminary descriptive data, given that the sample size (*n* = 9) does not meet the minimum threshold of 20 animals recommended by the ASVCP for preliminary reference interval studies, nor the minimum of 120 animals required for robust interval estimation (Friedrichs et al. 2012).

**Table 1:** Biochemical values of llamas (*Lama glama*) raised in a temperate climate and medium altitude in southwestern Paraná, Brazil

Parameters	Biochemical Values					
	Mean ± SD	Minimun	Maximun	CI 90% Upper	CI 90% Lower	CV
Uric-A (mg/dL)	0.3 ± 0.2	0.1	0.7	0.4	0.2	0.76
Alb (g/dL)	3.3 ± 0.6	2.3	4	3.7	3.0	0.17
ALT (U/L)	3 ± 2	1	6	4	2	0.68
AST (U/L)	160 ± 67	57	263	201	118	0.42
Ca (mg/dL)	8.9 ± 0.7	7.6	13.3	9.4	8.3	0.08
CK (U/L)	200 ± 163	29	397	309	91	0.81
Chol (mg/dL)	34 ± 15	16	63	43	25	0.45
Crea (mg/dL)	2.16 ± 0.42	1.6	2.78	2.44	1.88	0.19
ALP (U/L)	92 ± 47	44	162	131	54	0.51
Fe (mg/dL)	108 ± 44	61	181	135	81	0.41
GGT (U/L)	10.54 ± 5.27	3.51	19.5	13.81	7.28	0.50
LIP (U/L)	8 ± 5	4	18	12	5	0.54
PP (g/dL)	5.5 ± 1	3.7	6.7	6.1	4.9	0.18
Trig (mg/dL)	22 ± 6	13	32	26	18	0.26
Urea (mg/dL)	46 ± 10	29	61	53	40	0.21
Amyl	1339 ± 149	1122	1548	1448	1229	0.11
P (mg/dL)	8.8 ± 2.6	5.1	13	10.4	7.2	0.29
HDL Wide (U/L)	9 ± 4	4	17	12	6	0.48
Lac (mg/dL)	78.9 ± 28	48.6	125.7	96.2	61.5	0.36
Mag (mg/dL)	2.5 ± 0.4	2	2.9	2.7	2.3	0.14

Mean ± standard deviation (SD), minimum and maximum values, 90% confidence intervals (CI Upper and CI Lower), and coefficient of variation (CV) for serum biochemical parameters, including uric acid (Uric-A), albumin (Alb), alanine aminotransferase (ALT), aspartate aminotransferase (AST), calcium (Ca), creatine kinase (CK), total cholesterol (Chol), creatinine (Crea), alkaline phosphatase (ALP), iron (Fe), gamma-glutamyl transferase (GGT), lipides (LIP), plasmatic protein (PP), triglycerides (Trig), urea, amylase (Amyl), phosphorus (P), high-density lipoprotein (HDL), lactate (Lac) and magnesium (Mag)

**Table 2:** Blood gas values of llamas (*Lama glama*) raised in a temperate climate and medium altitude in southwestern Paraná, Brazil

Parameters	Blood Gas Values					
	Mean ± SD	Minimun	Maximun	CI 90% Upper	CI 90% Lower	CV
pH	7.269 ± 0.075	7.186	7.395	7.310	7.228	0.01
pCO2 (mmHg)	52.5 ± 1.5	51.3	55.7	53.3	51.7	0.03
cHCO3- (mmol/L)	25 ± 4.9	20.3	31.4	27.7	22.3	0.20
BE (ecf) (mmol/L)	-6.4 ± 1.9	-9.5	-3.7	-5.4	-7.4	-0.29
BE(b) (mmol/L)	-5.9 ± 1.7	-8.7	-3.5	-5.0	-6.9	-0.29
Na+ (mmol/L)	138 ± 6	128	149	141	135	0.04
Cl- (mmol/L)	127 ± 2	125	131	128	126	0.02
TCO2 (mmol/L)	25.3 ± 5.2	20.1	32.0	28.1	22.5	0.20
Glu (mg/dL)	146 ± 14	133	178	153	138	0.10
Lac (mmol/L)	4.84 ± 3.06	1.19	9.90	6.62	3.06	0.63
BUN (mg/dL)	26 ± 9	10	40	31	22	0.34
Crea (mg/dL)	2.39 ± 0.21	2.00	2.59	2.50	2.27	0.09

Mean ± standard deviation (SD), minimum and maximum values, 90% confidence intervals (CI Upper and CI Lower), and coefficient of variation (CV) for venous blood gas parameters, including pH, partial pressure of carbon dioxide (pCO₂), bicarbonate (HCO₃⁻), base excess (BE) in extracellular fluid (ecf) and blood (b), sodium (Na⁺), chloride (Cl⁻), total CO₂ (TCO₂), glucose (Glu), lactate (Lac), blood urea nitrogen (BUN), and creatinine (Crea)

The mean albumin concentration was 3.3 ± 0.6 g/dL, with a 90% confidence interval ranging from 3.7 to 3.0 g/dL. The mean levels of ALT and AST were 3 ± 2 U/L and 160 ± 67 U/L, respectively. The mean calcium (Ca) concentration was 8.9 ± 0.7 mg/dL. Regarding lipid metabolism, total cholesterol averaged 34 ± 15 mg/dL, while lipids presented a mean value of 8 ± 5 g/L. The mean triglyceride level was 22 ± 6 mg/dL.

Regarding renal function markers, creatinine concentration was 2.16 ± 0.42 mg/dL. Phosphorus levels were found to be 8.8 ± 2.6 mg/dL, and magnesium levels averaged 2.467 ± 0.346 mg/dL. The mean urea concentration was 46 ± 10 mg/dL, and uric acid averaged 0.3 ± 0.2 mg/dL. Hepatic enzyme activities showed that GGT levels averaged 10.54 ± 5.27 U/L, while FA reached a mean of 92 ± 47 U/L. Lactate concentration was 78.9 ± 28 mg/dL.

The blood gas parameters exhibit the mean pH value of 7.269 ± 0.075, while partial pressure of carbon dioxide of 52.5 ± 1.5 mmHg. The mean concentration of bicarbonate was 25 ± 4.9 mmol/L. Base excess (BE) in the extracellular fluid averaged − 6.4 ± 1.9 mmol/L, while in the blood, it was − 5.9 ± 1.7 mmol/L. Electrolyte analysis revealed mean sodium (Na+) concentration of 138 ± 6 mmol/L, while chloride (Cl-) levels averaged 127 ± 2 mmol/L.

The total carbon dioxide (TCO2) was 25.3 ± 5.2 mmol/L. The mean hematocrit value was 22 ± 2%, and hemoglobin concentration was 7.5 ± 0.8 g/dL. Glucose (Glu) concentration averaged 146 ± 14 mg/dL. Blood urea nitrogen (BUN) presented a mean value of 26 ± 9 mg/L, and creatinine concentration was 2.39 ± 0.21 mg/dL.

## Discussion

This study provides an analysis of biochemical and blood gas parameters in llamas (*Lama glama*) raised in a temperate climate at a medium altitude (595 m above sea level). The biochemical profile revealed that most values, as presented in Table 1, remained within the reference ranges described in the literature for the species (Fowler and Bravo 2010; Fowler and Zinkl 1989; Zaki et al. 2023).

Reference values established for domestic ruminants or other camelid species are often used as health indicators for llamas. However, individuals of the same species, whether in captivity or living freely, and originating from different geographic regions, may exhibit markedly different laboratory profiles (Antunes et al. 2018). The American Society for Veterinary Clinical Pathology (ASVCP) recognizes the need to establish population-specific reference intervals (Antunes et al. 2018).

The serum activities of ALT (3 ± 2 U/L), AST (160 ± 67 U/L), and CK (200 ± 163 U/L) showed marked individual variation among the animals. Foster et al. (2009) reported reference intervals of 0–14 U/L for ALT and 128–450 U/L for AST. Similarly, Zaki et al. (2023) reported mean values of 1.81 ± 0.34 U/L for ALT and 160.89 ± 7 U/L for AST in Egyptian llamas during the winter period. Thus, despite the individual variability observed in the present study, all ALT and AST values were within previously reported reference ranges for the species.

Although serum CK levels were higher than those reported in other studies, which range from 0 to 137 U/L (Foster et al. 2009; Zaki et al. 2023), this difference likely reflects characteristics of the studied population, as the animals were maintained under partial confinement (Kandeel et al. 2022). Therefore, handling procedures and physical restraint during sample collection may have contributed to the elevated CK levels (Zapata et al. 2003).

High glucose concentrations (146 ± 14 mg/dL) may reflect species-specific physiological and adaptive mechanisms in these animals, particularly differences in carbohydrate and enzymatic metabolismo (Cebra et al. 2001; Zaki et al. 2023; Zapata et al. 2003). Camelids characteristically exhibit higher blood glucose levels than domestic ruminants, with reported values ranging from 76 to 178.2 mg/dL (Foster et al. 2009; Fowler and Bravo 2010; Zaki et al. 2023), and display a relatively weak insulin response combined with slow cellular glucose uptake (Cebra et al. 2001; Foster et al. 2009).

The range of alkaline phosphatase activity observed in this study (92 ± 47 U/L) is consistent with values reported for the species in previous studies, which describe reference intervals of 27–132 U/L for adult South American camelids (Fowler and Bravo 2010; Fowler and Zinkl 1989), up to 0–610 U/L for llamas in general (Foster et al. 2009), and mean values of 109.06 ± 12.11 U/L for Egyptian llamas (Zaki et al. 2023).

This parameter presents considerable variation in a younger animals due the higher bone turnover rates. However, since the animals in this study were either adults or transitioning into adulthood, changes in this phase could indicate hepatic or bone alterations (Smith et al. 1998; Van Saun 2006).

Elevated blood urea concentrations (46 ± 10 mg/dL), accompanied by creatinine levels of 2.16 ± 0.42 mg/dL, were observed in the present study, whereas previous reports describe values ranging from 9 to 36 mg/dL for urea and from 0.9 to 2.8 mg/dL for creatinine in adult llamas (Foster et al. 2009; Fowler and Bravo 2010). These differences could be associated with the characteristics of the animals’ diet and the season in which sample collection was conducted (Zaki et al. 2023).

Previous studies in guanacos (*Lama guanicoe*) and llamas have reported seasonal variation in urea and creatinine concentrations related to protein intake, particularly during the winter period (Zapata et al. 2003; Zaki et al. 2023). In this context, Zaki et al. (2023) observed an increase in serum urea levels in llamas from 37.23 ± 3.28 to 56.32 ± 2.14 mg/dL during winter, which provides a comparative framework for interpreting the values obtained in the present study.

The animals in this study were managed under a semi-intensive system, which allows greater access to dietary protein. Their habitual diet consisted of *Panicum maximum* cv. Aruana and *Cynodon dactylon* (Florakirk) pasture, supplemented with wheat and oat grains provided in feed troughs. *Panicum maximum* cv. Aruana is recognized for its relatively high crude protein content (approximately 7.5–12% on a dry matter basis) and high digestibility (around 64%). This nutritional profile may partially account for the differences observed when compared with data from animals maintained under more restricted dietary conditions (Zaki et al. 2023).

Camelids are known for their efficient nitrogen conservation mechanisms, which enable them to thrive in protein-scarce environments by recycling urea through the gastrointestinal tract (Davies et al. 2007; Faustina and Willy 2024). Accordingly, llamas have been shown to exhibit lower renal urea excretion rates than other ruminants, such as sheep and goats (Davies et al. 2007; Dulphy et al. 1994).

The mineral profile observed in the studied animals revealed calcium concentrations (8.9 ± 0.7 mg/dL) similar to those previously reported for the species (7.6–10.9 mg/dL; Foster et al. 2009; Fowler and Bravo 2010). Calcium is not considered a reliable indicator of nutritional status due to its tightly regulated homeostasis; in contrast, phosphorus and magnesium concentrations are more closely associated with dietary intake (Van Saun 2006).

In the present study, phosphorus (8.8 ± 2.6 mg/dL) and magnesium (2.5 ± 0.4 mg/dL) levels were slightly higher than those generally reported in the literature. Fowler and Bravo (2010) described phosphorus values ranging from 1.6 to 11 mg/dL in adult specimens, whereas Foster et al. (2009) reported magnesium values between 1.94 and 2.58 mg/dL, and Zaki et al. (2023) reported mean phosphorus concentrations of 6.91 ± 0.44 mg/dL. These findings may be associated with the availability of high-quality forage during the rainy season, as both minerals are closely related to dietary content (Smith et al. 1998; Van Saun 2006).

Fowler and Bravo (2010) noted that serum inorganic phosphorus concentrations in llamas commonly show wide variability among reported reference values, with low levels frequently observed, as hypophosphatemia is a common clinical finding during prolonged periods of adverse weather when animals experience limited exposure to sunlight. In contrast, in the present study, llamas were raised under a semi-intensive system, with daily access to paddocks providing both natural light and shade. Additionally, in subtropical regions of Brazil, animals are exposed to relatively intense sunlight throughout most of the year, including during winter, when sampling was performed, which may have contributed to the phosphorus values observed.

Regarding blood gas analysis, the mean venous pH observed in this study was 7.269 ± 0.075. It is important to note that venous pH is naturally lower than arterial pH due to tissue metabolism (Hochachka et al. 1987; Viesselmann et al. 2018). This pH value, together with a mean pCO₂ of 52.5 ± 1.5 mmHg, differed from those previously reported for llamas (pH: 7.421 ± 0.048; pCO₂: 35.4 ± 6.25 mmHg) (Viesselmann et al. 2018).

The mean serum lactate concentration of 78.9 ± 28 mg/dL (approximately 8.8 mmol/L) was substantially higher than the reference values described for the species (7.26 ± 4.1 mg/dL) (Viesselmann et al. 2018). Several non-mutually exclusive factors may explain this finding. First, manual restraint without chemical sedation is a recognized source of acute lactate elevation in camelids. Physical exertion and stress-related catecholamine release during handling promote anaerobic glycolysis in skeletal muscle, transiently raising circulating lactate concentrations (Zapata et al. 2003; Cebra et al. 2001).

Second, the interval between venipuncture and serum biochemical analysis, even under refrigeration, can result in ongoing glycolysis in erythrocytes, artifactually increasing measured lactate (Kruse 2011). Third, point-of-care analyzers and standard wet-chemistry platforms differ in methodology, calibration, and reference ranges, making direct cross-method comparisons unreliable without prior validation (Viesselmann et al. 2018).

In addition, camelids demonstrate a notable tolerance to fluctuations in lactate and glucose concentrations, which has been attributed to their efficient metabolic regulation and ability to cope with environmental and handling-related stressors (Cebra et al. 2001; Faustina and Willy 2024). Nevertheless, the current study design does not allow attribution of elevated lactate levels to physiological adaptation. Therefore, this finding should be considered preliminary and requires independent validation through repeated sampling under controlled conditions, ideally including arterial blood gas analysis and comparison with standardized laboratory methods.

The biochemical and blood gas deviations observed in the present study are potentially consistent with previously described physiological characteristics of South American camelids, as well as with responses to the specific environmental and management conditions under which these animals were maintained (Fowler and Bravo 2010; Zaki et al. 2023).

The limited sample size (*n* = 9) precludes the establishment of reference intervals according to ASVCP guidelines, which recommend a minimum of 20 individuals for preliminary intervals and 120 for robust determination (Friedrichs et al. 2012). Therefore, the values reported here should be interpreted strictly as preliminary baseline data. In addition, several analytes exhibited high coefficients of variation, this degree of dispersion indicates marked inter-individual variability, reducing the reliability of mean values as representative descriptors of the population. Such variability may reflect biological heterogeneity, handling-related effects, or analytical factors, and reinforces the need for cautious interpretation and validation in larger cohorts.

It must be emphasized that the cross-sectional design, single time-point sampling, absence of a comparative control group (e.g., high-altitude or sea-level populations under equivalent management), and limited sample size collectively preclude any conclusion regarding stable physiological adaptation.

An additional limitation of the present study is the lack of detailed individual characterization of the animals, including body weight, body condition score, and reproductive status. These factors are known to influence biochemical and blood gas parameters in South American camelids and may contribute to inter-individual variability. Although all animals were clinically healthy and maintained under similar management and dietary conditions, the absence of these data precludes a more refined assessment of potential confounding effects.

These results improving the preliminary understanding of metabolic and respiratory physiology in llamas raised outside their native Andean habitats and underscore the importance of establishing species-specific reference intervals under different environmental and management conditions.

## Data Availability

My manuscript has no associated data. This study was conducted following ethical guidelines for animal research, ensuring minimal distress to the animals during handling and sample collection. All procedures were performed by trained veterinarians and in accordance with national and international animal welfare regulations. The study was approved by the institutional ethics committee (number 3749290824) of Federal University of Fronteira Sul.
